# Constitutive TRIM22 Expression in the Respiratory Tract Confers a Pre-Existing Defence Against Influenza A Virus Infection

**DOI:** 10.3389/fcimb.2021.689707

**Published:** 2021-09-21

**Authors:** Matthew Charman, Steven McFarlane, Joanna K. Wojtus, Elizabeth Sloan, Rebecca Dewar, Gail Leeming, Mohammed Al-Saadi, Laura Hunter, Miles W. Carroll, James P. Stewart, Paul Digard, Edward Hutchinson, Chris Boutell

**Affiliations:** ^1^MRC - University of Glasgow Centre for Virus Research, Glasgow, United Kingdom; ^2^Division of Protective Immunity and Division of Cancer Pathobiology, Children’s Hospital of Philadelphia, Philadelphia, PA, United States; ^3^Department of Pathology and Laboratory Medicine, University of Pennsylvania Perelman School of Medicine, Philadelphia, PA, United States; ^4^The Roslin Institute, University of Edinburgh, Midlothian, United Kingdom; ^5^Institute of Infection, Veterinary and Ecological Sciences, University of Liverpool, Liverpool, United Kingdom; ^6^Department of Animal Production, University of Al-Qadisiyah, Al-Diwaniyah, Iraq; ^7^National Infection Service, Public Health England, Porton Down, Salisbury, United Kingdom

**Keywords:** TRIM22, influenza, intrinsic immunity, antiviral defence, respiratory tract

## Abstract

The induction of antiviral effector proteins as part of a homeostatically controlled innate immune response to infection plays a critical role in limiting the propagation and transmission of respiratory pathogens. However, the prolonged induction of this immune response can lead to lung hyperinflammation, tissue damage, and respiratory failure. We hypothesized that tissues exposed to the constant threat of infection may constitutively express higher levels of antiviral effector proteins to reduce the need to activate potentially harmful innate immune defences. By analysing transcriptomic data derived from a range of human tissues, we identify lung tissue to express constitutively higher levels of antiviral effector genes relative to that of other mucosal and non-mucosal tissues. By using primary cell lines and the airways of rhesus macaques, we show the interferon-stimulated antiviral effector protein TRIM22 (TRIpartite Motif 22) to be constitutively expressed in the lung independently of viral infection or innate immune stimulation. These findings contrast with previous reports that have shown TRIM22 expression in laboratory-adapted cell lines to require interferon stimulation. We demonstrate that constitutive levels of TRIM22 are sufficient to inhibit the onset of human and avian influenza A virus (IAV) infection by restricting the onset of viral transcription independently of interferon-mediated innate immune defences. Thus, we identify TRIM22 to confer a pre-existing (intrinsic) intracellular defence against IAV infection in cells derived from the respiratory tract. Our data highlight the importance of tissue-specific and cell-type dependent patterns of pre-existing immune gene expression in the intracellular restriction of IAV from the outset of infection.

## Introduction

Exposure to viral pathogens is a constant threat to all living things and vertebrates have evolved multiple lines of defence to suppress infection, propagation, and transmission to new hosts. If viruses succeed in penetrating non-specific barrier defences, the activation of pattern recognition receptors (PRRs) by pathogen- and damage-associated molecular patterns (PAMPs and DAMPs, respectively) leads to the activation of innate immune defences. This culminates in the secretion of cytokines (including interferons) and the induction of interferon stimulated gene (ISG) products (Killip et al., 2015; Pulendran and Maddur, 2015). ISGs include a wide range of antiviral effector proteins, and their induced expression from low basal levels to high functional levels plays an important role in resolving infection (Schoggins et al., 2011; Durbin et al., 2013; Schoggins et al., 2014). However, the induction of this antiviral response necessitates pathogen detection. In the case of wild-type influenza A virus (IAV) this requires the detection of aberrant viral RNAs (vRNAs) produced during viral replication by PRRs for optimal induction (Tapia et al., 2013; Killip et al., 2017; Vasilijevic et al., 2017; Russell et al., 2019). Accordingly, the delayed activation of innate immune defences provides a window of opportunity for viral pathogens to express immunosuppressive genes, which can inhibit or dampen the efficacy of host immune defences (Hale et al., 2010; Hsu, 2018). A growing body of evidence suggests that this initial ‘gap’ in intracellular immunity is covered by constitutively expressed (continuously transcribed and translated) host factors that confer a pre-existing level of immune protection to susceptible cells, often referred to as intrinsic antiviral immunity (Bieniasz, 2004; Komatsu et al., 2016; Wu et al., 2018).

Effectors of intrinsic immunity are expressed at sufficient levels to confer protection from the outset of infection. As a result, they can restrict the initiation or progress of viral replication prior to the activation of PRRs and induction of innate immune defences (Brass et al., 2009; Fu et al., 2015; Patil et al., 2018). In some cases, the constitutive expression of these antiviral host factors can occur in a species-specific and cell-type dependent manner, which can influence host range and relative susceptibility to infection (Bieniasz, 2004; Wu et al., 2018; Bergantz et al., 2019). Although the importance of intrinsic immunity in the context of retrovirus and DNA virus infection is well established (Komatsu et al., 2016; Alandijany, 2019; Bergantz et al., 2019), evidence to support a biological role for intrinsic immunity during IAV infection has remained limited.

The TRIpartite Motif (TRIM) family of proteins comprise over 70 members that participate in a range of biological processes, including viral immune regulation (Van Tol et al., 2017; Van Gent et al., 2018; Koepke et al., 2020). Many TRIM proteins are upregulated in response to IFN stimulation and act as regulators or effectors of innate immunity. Other TRIM proteins are constitutively expressed to efficacious levels that can act to directly restrict infecting pathogens. Notably, several TRIM family members can confer protection against multiple viruses, suggesting they act as key effector molecules of the antiviral immune response (Koepke et al., 2020). For example, TRIM22 has been implicated in the cellular restriction of a broad range of viruses, including encephalomyocarditis virus [EMCV; (Eldin et al., 2009)], hepatitis B virus [HBV; (Gao et al., 2009)], hepatitis C virus [HCV; (Yang et al., 2016)], human immunodeficiency virus [HIV; (Barr et al., 2008; Turrini et al., 2015)], herpes simplex virus 1 [HSV-1; (Reddi et al., 2021)], and IAV (Di Pietro et al., 2013). Studies have shown TRIM22 to be an ISG, strongly upregulated by type-I and type-II IFNs (Hattlmann et al., 2012). Accordingly, TRIM22 has been shown to inhibit viral infection following its induced expression as an ISG by restricting the onset of viral transcription or targeted degradation of viral proteins (Hattlmann et al., 2012). With respect to IAV, TRIM22 has been reported to mediate the ubiquitination and proteasome-dependent degradation of NP, restricting IAV propagation as an induced effector protein of the type-I IFN response (Di Pietro et al., 2013; Pagani et al., 2018).

We hypothesized that localized patterns of immune gene expression at portals of viral entry may confer enhanced protection to cells susceptible to infection. We identify TRIM22 to be among several antiviral effector proteins to be constitutively expressed in the lung. Using primary cell lines of lung origin and the airways of rhesus macaques, we show the constitutive expression of TRIM22 to occur independently of interferon-stimulation or viral infection. We demonstrate endogenous levels of TRIM22 to be sufficient to restrict the initiating cycle of human and avian IAV infection independently of IFN-mediated innate immune defences. Thus, we identify TRIM22 to confer a pre-existing (intrinsic) intracellular immune barrier to IAV infection. Transcriptomic analysis revealed TRIM22 to be one of a large group of antiviral host factors that are downregulated in many laboratory-adapted cell lines commonly used for virus research. Collectively, our data demonstrate tissue-specific and cell-type dependent patterns of pre-existing immune gene expression to play a previously unappreciated role in the cellular restriction of IAV from the outset of infection.

## Materials and Methods

### Animals and Ethics

No animals were directly subjected to experimentation as part of this scientific study. All animal tissues were obtained from material produced in previously described experiments (Marriott et al., 2016) with permission from Public Health England (PHE). Procedures associated with this earlier study were approved by the PHE Ethical Review Committee (Porton Down, UK), authorized under UK Home Office project licence 30/3083, and performed according to UK Animal (Scientific procedures) Act 1986. 3- to 3.5-year-old male cynomolgus macaques (*Macaca fascicularis*) were housed in socially compatible groups of 4 in cages with a floor of deep litter for foraging and a range of toys, puzzles, and DVDs for stimuli. The colony was regularly screened and shown to be free from Herpes B virus, Simian T-cell Lymphotropic virus, Simian retrovirus, Simian immunodeficiency virus, *Mycobacterium tuberculosis*, Salmonella sp., and influenza H1N1pdm and H3N2 viruses. Animals were sedated by intramuscular injection prior to infection with ketamine hydrochloride (Marriott et al., 2016). Animals were infected with 6x10^6^ PFU of IAV (A/California/04/2009 (H1N1); Cal) *via* intra-tracheal inoculation and euthanized 5 days post-infection (Marriott et al., 2016). Animals were monitored for health and wellbeing throughout the study (Marriott et al., 2016). Tissues were collected at necropsy and fixed in 10% neutral buffered formalin, processed to paraffin wax, and cut into 5-6 µm sections.

### Quantitative Histopathology of Cynomolgus Macaque Tissue Sections

Deparaffinised and rehydrated tissue sections were processed for epitope retrieval using EnVision FLEX target retrieval solution (Agilent, K8004), as per manufacturer’s instructions. Samples were processed and stained for TRIM22 (Proteintech, 13744-1-AP; 1/50 dilution) and FLEX haematoxylin (Agilent, K8008) using a Autostainer Link 48 (Agilent), as per manufacturer’s instructions. TRIM22 antibody specificity was validated by staining sectioned MRC5 (positive control) or A549 (negative control) cell pellets. Tissue sections were independently assessed for TRIM22 expression by a qualified pathologist. Automated quantitation of TRIM22 expression levels was performed using whole-slide scans and Image-Pro Premier (Media Cybernetics), as previously described (Leeming et al., 2015; Akram et al., 2018).

### Quantitative Histopathology of Human Tissue Sections

IHC data from anonymized human bronchus and nasopharynx tissues stained for TRIM22 were obtained from the Human Protein Atlas [HPA; http://www.proteinatlas.org; (Uhlen et al., 2010; Uhlen et al., 2015)] under a Creative Commons Attribution-ShareAlike 3.0 International License. The original images consulted were TRIM22 Bronchus (http://www.proteinatlas.org/ENSG00000132274-TRIM22/tissue/bronchus#img) and TRIM22 Nasopharynx (http://www.proteinatlas.org/ENSG00000132274-TRIM22/tissue/nasopharynx#img).

### RNA-Seq Analysis of Human Cell Lines and Tissues

RNA-Seq data for human cell lines (HBEC3-KT, A549, HEK 293, and HeLa) and anonymized human tissue (lung, small intestine, esophagus, colon, liver, skin, and kidney) were obtained from Human Protein Atlas [HPA; http://www.proteinatlas.org, version 18.1; (Uhlen et al., 2010; Uhlen et al., 2015)] or Genotype-Tissue Expression project [GTEx; https://gtexportal.org/home/, V7; (Carithers et al., 2015)] supported by the Common Fund of the Office of Director of the National Institutes of Health, and by NCI, NHGRI, NHLBI, NIDA, NIMH, and NINDS under Creative Commons Attribution-ShareAlike 3.0 International License. Principle Component Analysis (PCA) cluster plots were generated using kmeans and clusplot packages and prcomp function in R (https://www.r-project.org). Heatmaps were generated using Prism 8 (GraphPad) or pheatmap (v1.0.12) and cluster (v2.0.7-1) package in R.

### Cells, Viruses, and Drugs

Primary human foetal lung fibroblast (MRC5) cells were purchased from the European Collection of Authenticated Cell Cultures (ECACC; 05072101). MRC5t cells are immortalized MRC5 cells expressing the catalytic subunit of human telomerase (hTERT), and were generated as previously described (Smith et al., 2013). MRC5 and MRC5t cells were cultured in Dulbecco’s Modified Eagle Medium (DMEM; Life Technologies; 41966) supplemented with 10% foetal bovine serum (FBS; Life Technologies; 10270), 100 U/ml of penicillin and 100 μg/ml of streptomycin (P/S; Life Technologies; 15140-122), and 1× non-essential amino acids (NEAA; Life Technologies 11140-035). MRC5t cells were supplemented with 5 μg/mL hygromycin B (Thermo Fisher Scientific; 10687010) to maintain hTERT expression. MRC5t cells were transduced with lentiviruses to express short hairpin (sh) RNAs based on the 19-mer sequences; non-targeting control (shCtrl; 5’-ttatcgcgcatatcacgcg-3’) or TRIM22 targeting (shTRIM22 clone B7 [3’ UTR]; 5’-TATTGGTGTTCAAGACTAT-3’, clone B8; 5’-CTGTACGCACCTGCACATT-3’, clone B9; 5’-GTGTCTTCGGCTGCCAATA-3’), as previously described (Everett et al., 2006). Pooled, stably transduced cells were maintained in growth media supplemented with 0.5 µg/ml puromycin (Sigma-Aldrich; P8833). Primary human bronchial epithelial (HBEp) cells were purchased from Sigma-Aldrich (502-05a). hTERT and CDK4 immortalized human bronchial epithelial cells (HBEC3-KT) were purchased the Hamon Center for Therapeutic Oncology Research [UT Southwestern Medical Center; (Ramirez et al., 2004)]. Cells were cultured according to supplier guidelines. Madin Darby Canine Kidney (MDCK; a gift from Ben Hale University of Zurich), human lung adenocarcinoma epithelial (A549; PHE Culture Collections, 86012804), human embryonic kidney (HEK 293T; a gift from Roger Everett MRC-UoG CVR) and human cervical carcinoma (HeLa [a gift from Juergen Hass University of Edinburgh] or HEp2 [a gift from Roger Everett MRC-UoG CVR]) cells were cultured in DMEM with 10% FBS and P/S. All cells were maintained at 37°C in 5% CO_2_. IAV strains A/WSN/1933(H1N1) (WSN), A/Puerto Rico/8/1934(H1N1) (PR8), A/Udorn/307/1972(H3N2) (Udorn), and A/California/04/2009(H1N1) (Cal) were propagated in MDCK cells. A/Duck/Singapore/5/1997(H5N3) (Duck H5N3) and A/Chicken/Italy/1067/1999(H7N1) (Chicken H7N1) were propagated in embryonated chicken eggs. WSN titres were calculated by immunocytochemistry (ICC) plaque assay, as described below. PR8, Udorn, Duck H5H3 and Chicken H7N1 titres were calculated based on focus forming units (FFU), as described below. Cells were interferon stimulated by the addition of 100 IU/ml recombinant interferon-β (IFN-β; Merck, 407318) to the growth media for 24 h. Ruxolitinib (Ruxo; Selleckchem; S1378) was prepared in DMSO and used at the concentrations indicated.

### Plaque, FFU, and Virus Yield Assays

For plaque assays, cells were seeded at 2 × 10^5^ cells/well in 12-well dishes and incubated for a minimum of 16 h prior to infection. Cells were infected with serial dilutions of virus for 1 h at 37°C prior to overlay with conditioned growth medium supplemented with 1.2% Avicel (Biopolymers; RC-591), 0.1% sodium bicarbonate (Life Technologies; 25080-060), and 0.01% DEAE Dextran (Sigma-Aldrich; D9885). Cell monolayers were processed for ICC staining at 24 to 72 hpi depending on virus replication kinetics [as previously described (Conn et al., 2016)] or stained with Giemsa stain (VWR; 35086). Relative plaque titre was calculated as the plaque titre of a virus stock under the indicated condition divided by its titre under a control condition. For FFU assays, 1 x 10^5^ MRCt cells were seeded onto 13mm glass coverslips and incubated for 16 h prior to infection. Viral stocks were serially diluted and used to infect MRC5t cells for 1 h at 37°C prior to overlay with conditioned growth medium. Cells were fixed at 8 hpi, immunostained for the viral protein NP, and analyzed by immunofluorescent confocal microscopy. The total number of cells and number of NP positive cells imaged was determined using ImageJ from a minimum of 5-fields of view per dilution, and the % of NP positive cells calculated. The dilution resulting in closest to 50% infected cells was selected for calculation of FFU and adjusted for the Poisson distribution, as previously described (Hutchinson et al., 2008). Viral titres were determined from a minimum of 3 independent titrations. For virus yield assays, cells were infected with IAV (WSN) at the indicated multiplicity of infection (MOI) for 1 h at 37°C, washed twice with PBS, then overlaid with growth medium. Supernatants were collected at the indicated time points post-infection and the released virus was titred by plaque assay in MDCK cells. Plaque diameters were measured using an automated Celigo imaging cytometer (Nexcelom biosciences), as per the manufacturer’s instructions.

### Antibodies

Polyclonal antibodies were used to detect TRIM22 (Sigma-Aldrich; HPA003575), Mx1 (Santa Cruz; sc-50509), GBP1 (Proteintech; 15303-1-AP), IFIH1 (Proteintech; 21775-1-AP), TLR3 (Proteintech; 17766-1-AP), and actin (Sigma-Aldrich; A5060). IAV hybridoma antisera were used to detect NP, M1, and NS1, as previously described (Turnbull et al., 2016). Monoclonal antibodies were used to detect UBA7 (AbCam; ab133499), Actin (DSHB; 224-236-1), IFITM1 (Proteinech; 60074-1g), and IAV NP (AbCam; ab20343). Secondary antibodies were Alexa 488 and 555 donkey anti-mouse and -rabbit (Invitrogen; A21202, A21206, and A31572), DyLight 680- or 800-conjugated anti-rabbit (Thermo Fisher Scientific; 35568 and SA5-35571), and peroxidase conjugated anti-mouse (Sigma-Aldrich; A4416).

### Immunofluorescence Confocal Microscopy

1 × 10^5^ cells were seeded on 13 mm glass coverslips in 24-well dishes and incubated for a minimum of 16 h prior to experimentation. After treatment, cell monolayers were fixed, permeabilized, and immunostained at the indicated time points, as previously described (Conn et al., 2016). Nuclei were stained with DAPI (Sigma-Aldrich, D9542). Coverslips were mounted on glass slides using Citiflour AF1 mounting medium (AgarScientific; R1320) and sealed with nail enamel. Samples were examined with a Zeiss LSM 880 or LSM 710 confocal microscope with 405, 488, 543, and 633-nm laser lines. Images were captured under a Plan-Apochromat 63×/1.4 oil immersion or Plan-Neofluar 20×/0.5 air objective lenses. The proportion of IAV NP antigen positive cells was calculated from a minimum of five wide field images, imaging more than 1000 cells per coverslip per condition. The proportion of NP positive cells was determined and the fold increase in NP positive cells between IAV infected shTRIM22 and shCtrl MRC5t cells calculated for each biological repeat.

### Western Blotting

Cells were seeded at 2 × 10^5^ cells/well in 12-well dishes and incubated for a minimum of 16 h prior to experimentation. Treated or infected cell monolayers were washed twice in PBS and whole cell lysates (WCLs) collected in Laemmli buffer. Proteins were resolved on NOVEX NU-PAGE (4-12%) Bis-Tris gels (Invitrogen; NP0322), transferred onto 0.22 µm nitrocellulose membranes (Amersham; 15249794), and probed by western blotting, as previously described (Conn et al., 2016). Membranes were imaged using an Odyssey Infrared Imager (Li-Cor). Band intensities were quantified using Image Studio Software (Li-Cor). Individual values were normalized to their respective actin loading controls prior to normalization to their respective control group (as indicated).

### qRT-PCR

For viral or cellular mRNA quantitation, cells were seeded at 2 × 10^5^ cells/well in 12-well dishes and incubated for a minimum of 16 h prior to experimentation. Treated or infected cell monolayers were washed once in PBS prior to RNA extraction using an RNAeasy Plus Kit (Qiagen; 74134). mRNA was reverse transcribed (RT) using the TaqMan Reverse Transcription Reagents kit (Life Technologies; N8080234) with oligo (dT) primers. Samples were analysed in triplicate using the TaqMan Fast Universal PCR Master Mix (Life Technologies, 4352042) and TaqMan *GAPDH* (4333764F), *TRIM22* (Hs01001179_m1), *Mx1* (Hs00895608_m1) or *ISG15* (Hs01921425_s1) specific primer-probe (FAM/MGB; Thermo Fisher Scientific) mixes or custom IAV (*NP*, *M1*, *NS1*/*NEP*) primer-probes mixes (**Supplemental Table S5**). The ΔΔCt method was used to normalize transcript levels to those of *GAPDH* mRNA. For vRNA analysis, cells were seeded at 4 × 10^5^ cells/well in 6-well plates and incubated for a minimum of 16 h prior to experimentation. For vRNA analysis, total RNA was isolated from nuclear pellets using an RNAeasy Plus Kit. An IAV segment 7 specific primer (5’-AGCCGAGATCGCACAGAGACTT-3’) was used for reverse transcription, as previously described (Vester et al., 2010). Samples were analysed in triplicate using the M1 primer-probe mix relative to a synthetic segment 7 (M) vRNA reference standard. The segment 7 vRNA standard was produced as previously described (Vester et al., 2010). Briefly, vRNA was extracted from infected MDCK cells using the QIAamp Viral RNA Mini kit (Qiagen, 52904) and extracted RNA was reverse transcribed using the Uni12 universal IAV segment primer (5’-AGCAAAAGCAGG-3’) and TaqMan Reverse Transcription Reagent kit. The cDNA was used as a template to amplify the IAV segment 7 ORF, incorporating a T7 promoter sequence that was used to generate synthetic segment 7 vRNA using the TranscriptAID T7 high yield transcription kit (Thermo Fisher Scientific; K0441). Synthetic vRNA was purified using an RNAeasy column and used as a reference standard for reverse transcription and qRT-PCR analysis.

## Results

### Human Lung Tissue Is Enriched for Constitutive ISG Expression

As the respiratory tract is a major portal for virus entry, we hypothesized that cells in the respiratory mucosa might express antiviral effector proteins to higher levels than cells in tissues at less exposed locations, thereby creating localized patterns of pre-existing antiviral defence. We reasoned that if these pre-existing effector proteins were constitutively expressed to efficacious levels prior to infection, they will be amongst the most abundantly expressed antiviral proteins in healthy human tissue.

**Figure 1:**
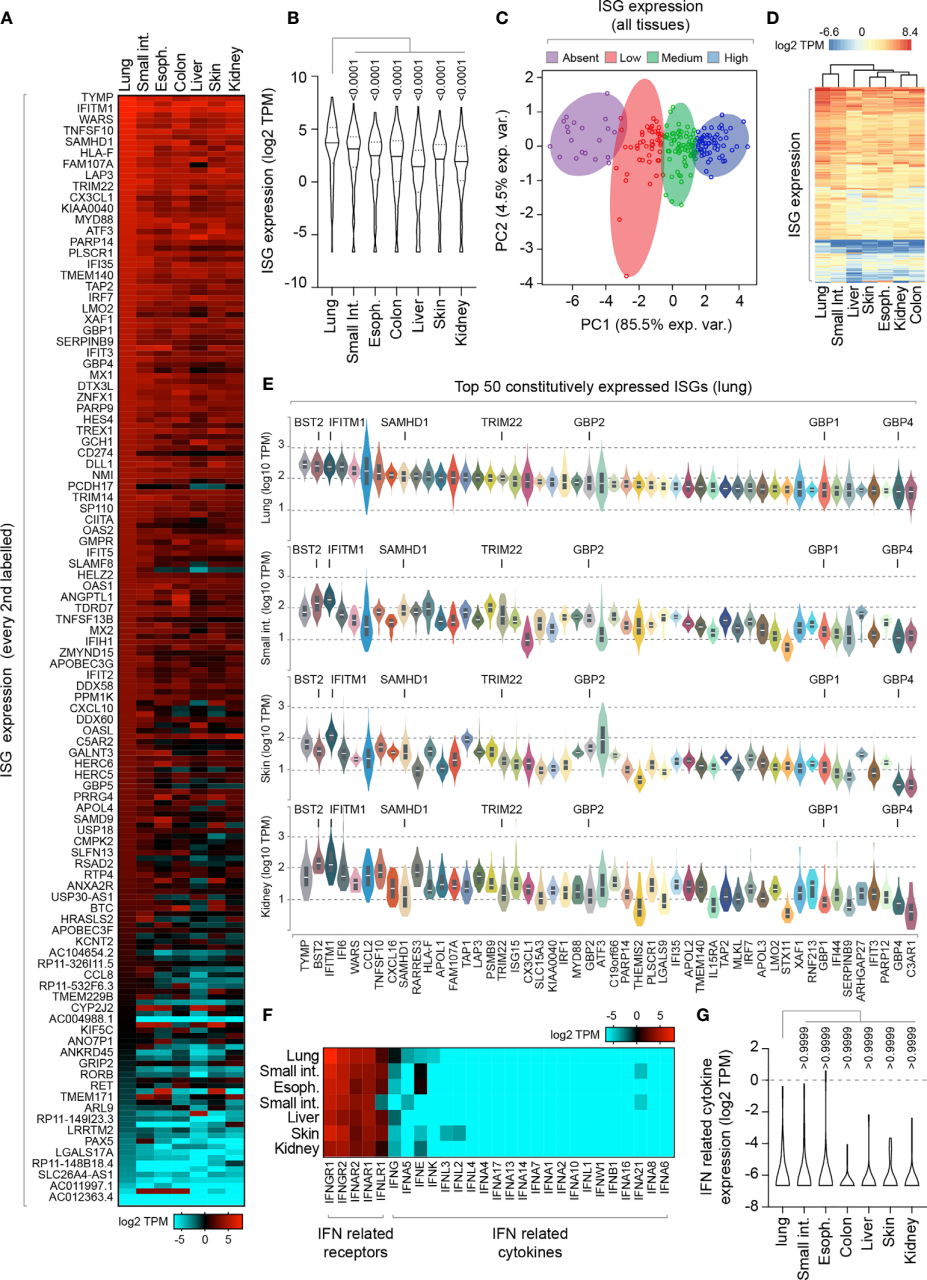
Lung tissue is enriched for constitutive ISG expression relative to other mucosal and non-mucosal tissues. **(A)** Heat map showing the constitutive expression profile (log2 transcripts per million; TPM) of 200 ISGs across a range of human mucosal and non-mucosal tissues; lung (n=427), small intestine (Int.; terminal ileum; n=137), esophagus (Espho.; mucosa; n=407), colon (sigmoid; n=233), liver (n=175), skin (suprapubic; n=387), and kidney (cortex; n=45). Every second ISG labelled (individual values shown in **Supplemental Table S1A**). **(B)** Violin plot showing tissue ISG expression profiles (as in **A**). Horizontal solid lines; median ISG expression per tissue. Horizontal dotted lines; 5^th^ and 95^th^ percentile range per tissue. **(C)** Principle component (PC) analysis of ISG expression levels across all tissues (as in **A**). **(D)** Heatmap showing clustered relationship of ISG expression levels between different tissues (as in **A**). **(E)** Violin plots showing the top 50 constitutively expressed ISGs in the lung relative to small intestine, skin, and kidney tissues. White line; median. Box; 5^th^ and 95^th^ percentile range. **(F)** Heat map showing the expression profile (log2 TPM) of IFN-related receptors and cytokines per tissue (as in **A**; values shown in **Supplemental Table S1B**). **(G)** Violin plot showing IFN-related cytokine expression profile (as in **F**). Horizontal solid lines; median. Horizontal dotted lines; 5^th^ and 95^th^ percentile range. **(E, F)** Paired one-way ANOVA (Friedman multiple comparison test); p-values shown. RNA-Seq data adapted under creative commons licence from GTEx portal [https://gtexportal.org/home/; (Carithers et al., 2015)].

To investigate, we began by analyzing the constitutive expression profile of 200 ISGs across a range of human tissues derived from a mixture of cell types [Genotype-Tissue Expression (GTEx) project; (Carithers et al., 2015; Shaw et al., 2017)]. We analysed RNA-seq data from mucosal (lung, esophagus, colon, and small intestine) and non-mucosal (liver, skin, and kidney) tissues (**Figure 1** and **Supplemental Table S1A**). We identified tissue-specific profiles of constitutive ISG expression, with lung tissue expressing the highest level of ISG expression relative to all other tissues examined (**Figures 1A, B**). Principle component analysis (PCA) demonstrated that not all ISGs were constitutively expressed or expressed to equivalent levels (**Figure 1C**), with lung tissue sharing the highest degree of ISG profile similarity to that of the small intestine (**Figure 1D**). Notably, many of the top 50 constitutively expressed ISGs in the lung were known antiviral host factors, including BST2, IFITM1, SAMHD1, TRIM22, and GBP family members (**Figure 1E**) (Schoggins et al., 2011). Importantly, IFN transcript levels were either not detectible or extremely low (< 1 TPM; **Figures 1F, G** and **Supplemental Table S1B**), indicating that these tissues were not constitutively expressing IFN at the point of RNA extraction. While we cannot rule out a role for tonic-IFN signalling or host microbiota in the tissue-dependent enrichment of ISGs (Tovey et al., 1987; Gough et al., 2012; Liu et al., 2018; Bradley et al., 2019), our analysis identifies enriched levels of ISG expression in the lung that could confer enhanced protection upon pathogen entry.

### TRIM22 Is Constitutively Expressed at High Levels in the Respiratory Tract Independently of Immune Stimulus or Virus Infection

**Figure 2:**
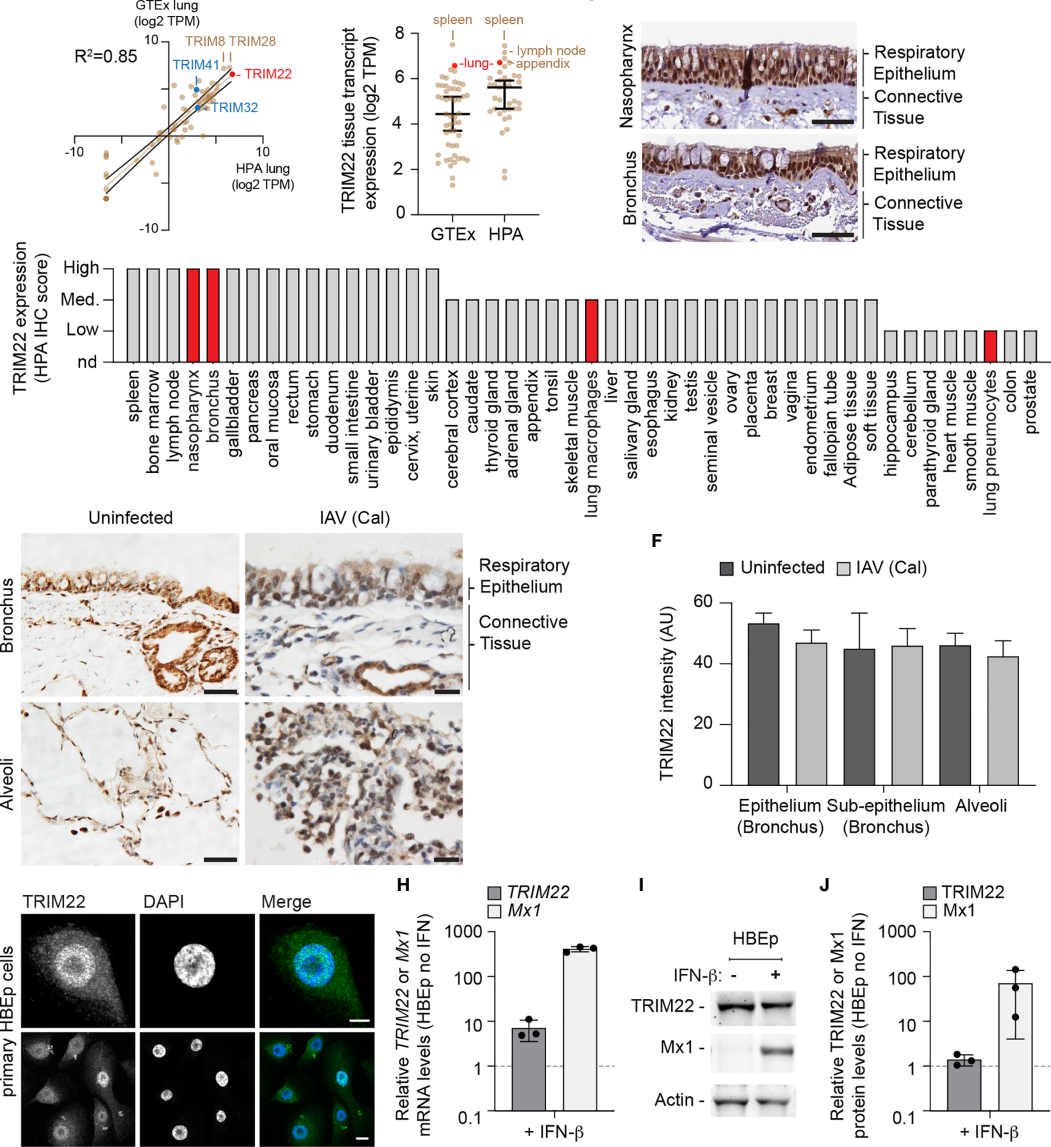
TRIM22 is constitutively expressed in the respiratory tract independently of IAV infection. **(A)** Scatter plot showing TRIM family member expression levels (log2 TPM) in human lung tissue derived from two independent data sources (GTEx and HPA, respectively; values shown in **Supplemental Table S2A**). Solid brown line; linear regression, R-squared (R^2^ = 0.85). Solid black lines; 95% confidence interval. **(B)** Scatter plot showing TRIM22 expression levels (log2 TPM) across a range of tissues (as in **A**; values shown in **Supplemental Table S2B**). Black line: median TRIM22 transcript expression; whisker: 5^th^ to 95^th^ percentile range. **(C)** Histological sections of human respiratory epithelium from the upper (nasopharynx; patient ID 3624) and lower (bronchus; patient ID 3987) airway. TRIM22 is labelled by immunohistochemistry (IHC; brown). Tissues were counterstained with haematoxylin and eosin. Scale bar 50 µm. **(D)** Quantitation of TRIM22 IHC staining across a range of human tissues. Red bars highlight TRIM22 expression in cells of lung origin. **(A–D)** Data adapted under creative commons license from GTEx portal [https://gtexportal.org/home/; (Carithers et al., 2015)] and Human Protein Atlas [HPA; https://www.proteinatlas.org; (Uhlen et al., 2010; Uhlen et al., 2015)]. **(E)** Histological sections of uninfected or intra-tracheal influenza A virus [IAV; A/California/04/09(H1N1), Cal] infected (6x10^6^ PFU) cynomolgus macaques at 5 days post-infection. Tissue sections of respiratory epithelium derived from the bronchus or alveoli were stained for TRIM22 by IHC (brown) and counterstained with haematoxylin. Scale bars; 50 and 20 µm (left and right panels, respectively). **(F)** Automated quantitation of TRIM22 IHC staining in uninfected or infected cynomolgus macaque respiratory tissue from whole-slide scans (as in **E**). Means and SD from four uninfected and three infected animals shown. **(G)** Confocal images of primary human bronchial epithelial (HBEp) cells stained for TRIM22 by indirect immunofluorescence (green). Nuclei stained with DAPI (blue). Scale bars; 10 µm (top panel) and 20 µm (bottom panel). **(H–J)** Primary HBEp cells were treated with (+) or without **(-)** IFN-β (100 IU/ml) for 24 h. **(H)** qRT-PCR of *TRIM22* and *Mx1* mRNA transcript levels in control or IFN treated HBEp cells. Values normalized to no IFN treatment (dotted line); n=3, means and SD shown. **(I)** Western blots of HBEp whole cell lysates (WCLs) probed for TRIM22 and Mx1 expression. Actin is shown as a loading control. **(J)** Quantitation of western blots (as in **J**). Values normalized to actin and expressed relative to no IFN treatment (dotted line); n=3, means and SD shown.

To further explore our observation that lung tissue shows enriched levels of antiviral gene expression, we examined the expression profile of TRIM family members which include both interferon-stimulated and constitutively expressed antiviral effector proteins (Van Tol et al., 2017; Van Gent et al., 2018; Koepke et al., 2020). We compared the profiles of TRIM expression from two independently-derived lung transcriptome data sets [GTEx project and Human Protein Atlas (HPA); (Uhlen et al., 2010; Carithers et al., 2015; Uhlen et al., 2015)]. Our analysis identified TRIM22 to be one of the most abundantly expressed TRIM family members in the lung (**Figure 2A**, red circle; **Supplemental Table S2A**) independently of detectible levels of IFN expression (**Supplemental Table S1B**). The expression of TRIM22 was comparable to that of TRIM8 and TRIM28, and higher than that of TRIM32 and TRIM41, which have been reported to be intrinsic antiviral effectors of IAV replication (Fu et al., 2015; Patil et al., 2018). Notably, the relative expression levels of TRIM8, 28, 32, and 41 are not influenced by IFN stimulation (Shaw et al., 2017). The high levels of constitutive TRIM22 expression was surprising, since this level of expression has been previously reported to require viral infection or IFN stimulation (Hattlmann et al., 2012; Di Pietro et al., 2013). We therefore investigated if this profile of constitutive TRIM22 expression was specific to lung tissue. We found TRIM22 expression to be enriched in the spleen, lymph node, appendix, and lung relative to other tissues (**Figure 2B**, red circle; **Supplemental Table S2B**). Analysis of HPA immunohistochemistry (IHC) expression records confirmed high levels of constitutive TRIM22 protein expression in the nasopharynx and bronchus (**Figures 2C, D**). Together, these data demonstrate that TRIM22 is expressed to high levels in the respiratory tract relative to other tissues or TRIM family members.

As we had identified TRIM22 to be amongst the top 50 constitutively expressed ISGs in the lung, we next analysed how TRIM22 expression in the respiratory tract responded to viral infection. As mice lack an orthologue to human TRIM22 (https://www.ncbi.nlm.nih.gov/homologene/?term=trim22), we examined respiratory tissue derived from cynomolgus macaques (*Macaca fascicularis*), whose TRIM22 has 92% amino acid identity to human TRIM22. Using a validated TRIM22 antibody (HPA; see below), we could detect TRIM22 in the respiratory tract of uninfected macaques, specifically in epithelia of the airways, sub-mucosal glands, alveoli, and in alveolar macrophages (**Figure 2E**, uninfected). To determine whether TRIM22 expression increased during infection, we compared healthy macaques with those infected with IAV (A/California/04/2009 (H1N1); Cal). These macaques were infected as part of a previous study and were confirmed to be undergoing an innate immune response at the point of euthanasia (Marriott et al., 2016). Automated staining and quantitation of sectioned samples demonstrated TRIM22 expression did not increase in the respiratory tract of IAV infected macaques relative to the control group (**Figures 2E, F**).

As the expression of TRIM22 in the respiratory tract contrasted with its reported expression in many laboratory-adapted cell lines and primary lymphocytes (Mo et al., 2007; Hattlmann et al., 2012; Di Pietro et al., 2013), we examined TRIM22 expression in primary human bronchial epithelial (HBEp) cells. TRIM22 was readily detectable in unstimulated cells by indirect immunofluorescence (**Figure 2G**), showing the same predominantly nuclear localization observed by IHC in respiratory epithelia (**Figures 2C, E**). We next examined how TRIM22 expression in HBEp cells responded to IFN stimulation. The addition of IFN-β caused an intense upregulation of the ISG Mx1 at both the transcript and protein level. In contrast, TRIM22 expression in the same cells was only moderately increased at the transcript level and not substantially increased at the protein level (**Figures 2H–J**). Together, these data demonstrate TRIM22 to be constitutively expressed in primary cells of lung origin independently of infection or immune stimulation. Collectively, these data suggest that endogenous levels of TRIM22 could potentially act as a pre-existing antiviral defence against respiratory airway infection.

### Constitutive Expression of TRIM22 Occurs in a Cell-Type Dependent Manner

**Figure 3:**
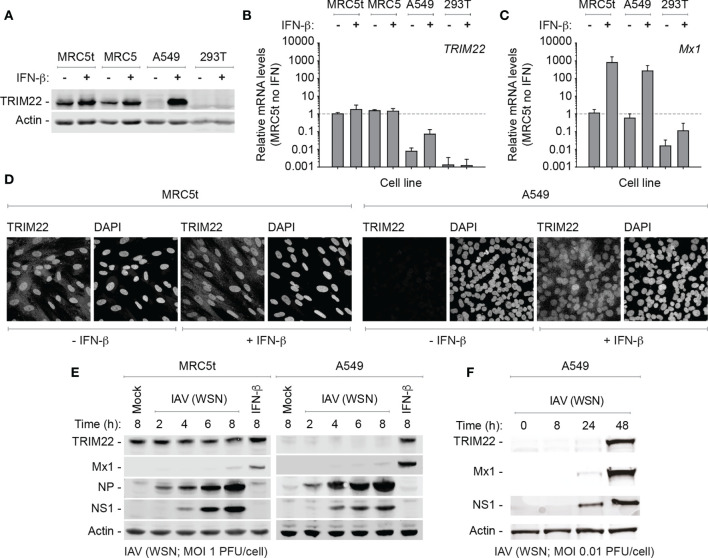
TRIM22 is differentially expressed in a cell-type dependent manner. **(A–C)** Primary and hTERT immortalized human lung fibroblast (MRC5 and MRC5t, respectively), lung adenocarcinoma epithelial (A549), and SV40-transformed human kidney epithelial (HEK 293T; 293T) cells were treated with (+) or without (-) IFN-β (100 IU/ml) for 24 h. **(A)** Western blots of WCLs probed for TRIM22 expression. Actin is shown as a loading control. **(B, C)** qRT-PCR quantitation of *TRIM22* and *Mx1* mRNA transcript expression levels, respectively. Values normalized to MRC5t cells without IFN treatment (dotted line); n=3, means and SD shown. **(D)** Confocal microscopy images of MRC5t and A549 cells with or without IFN treatment (as in **A**). TRIM22 was labelled by indirect immunofluorescence. Nuclei were stained with DAPI. **(E)** MRC5t and A549 cells were mock-treated, IFN- β (100 IU/ml) stimulated or infected with IAV (A/WSN/1933(H1N1), WSN) at a MOI of 1 PFU/cell (based on MDCK titres) for the indicated times (hours; h). WCLs were analyzed by western blots for TRIM22, Mx1, and viral protein (NP and NS1) expression. Actin is shown as a loading control. **(F)** A549 cells were infected with IAV (WSN; MOI of 0.01 PFU/cell based on MDCK titres) and harvested at the indicated times prior to western blotting (as in **E**).

While we could demonstrate constitutive levels of TRIM22 expression in primary HBEp cells, these cells are challenging to maintain in quantities suitable for functional studies due to the onset of cellular senescence. Accordingly, to identify a tractable cell line that maintained the constitutive profile of endogenous TRIM22 expression observed in the airway, we screened a panel of human cell lines for TRIM22 expression with or without IFN-β stimulation. In all transformed cell lines examined (HEK 293T, HeLa, HEp2, and A549), TRIM22 was either absent or only detected following IFN-β stimulation, as observed for the ISG Mx1 (**Figures 3A–D** and **Figure S1A, B**). This was surprising and indicated that infection studies that utilized such cell lines would not capture the potential antiviral properties of endogenous TRIM22 observed in the respiratory tract (**Figures 1**, **2**). In contrast, both primary and hTERT (human telomerase reverse transcriptase) immortalized human lung fibroblasts (MRC5 and MRC5t, respectively) retained constitutive levels of TRIM22 expression independently of IFN-stimulation (**Figures 3A–D**). As single-cell transcriptomics experiments have shown lung fibroblasts to be susceptible to IAV infection *in vivo* (Steuerman et al., 2018), we chose MRC5t cells as a model cell line to study the antiviral properties of constitutive TRIM22 expression against IAV infection.

As IAV infection is known to activate innate immune pathways that lead to the induction of ISGs, we began by investigating TRIM22 expression levels over a time course of IAV infection (A/WSN/1933(H1N1), WSN; MOI 1 PFU/cell) in MRC5t and A549 cells, which constitutively or inducibly express TRIM22, respectively (**Figures 3A, B**). Relative to IFN stimulation alone, ISG Mx1 expression was barely detectible at 8 hpi in both cell types (**Figure 3E**). In contrast, TRIM22 was continuously expressed throughout the time course of infection in MRC5t cells and remained undetectable in A549 cells, although readily detected upon IFN stimulation (**Figure 3E**). To investigate the conditions required for the induction of TRIM22 in A549 cells, we infected cells (WSN; MOI 0.01 PFU/cell) over an extended time course of infection to allow for multiple cycles of IAV replication that could stimulate the activation of IFN-mediated immune defences (Tapia et al., 2013; Killip et al., 2017; Russell et al., 2019). Under these conditions, both TRIM22 and Mx1 were robustly detected by 48 hpi (**Figure 3F**), indicative of the activation of IFN-mediated innate immune defences and induction of ISG expression (Di Pietro et al., 2013; Russell et al., 2019). We conclude that the constitutive expression of TRIM22 occurs in a cell-type dependent manner, with many laboratory-adapted cell lines requiring prolonged periods of infection or immune stimulation to produce detectible levels of TRIM22 expression. Thus, cell types which constitutively express TRIM22 might confer a pre-existing antiviral defence to respiratory airway infection.

### Constitutively Expressed TRIM22 Provides a Level of Pre-Existing Defence Against IAV Infection

**Figure 4:**
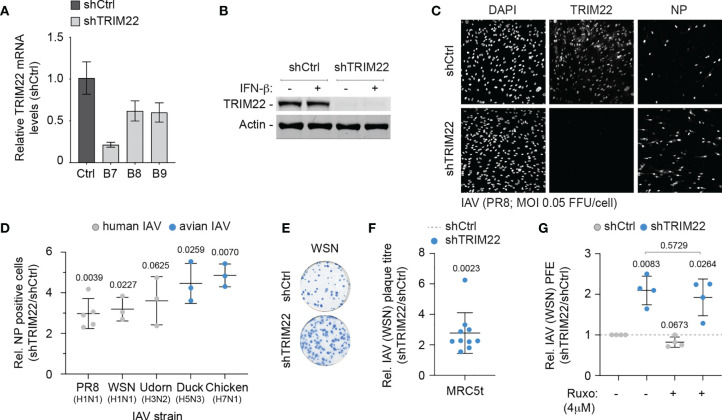
Constitutive TRIM22 expression confers pre-existing antiviral immunity. MRC5t cells were stably transduced to express non-targeting control (shCtrl) or TRIM22-targeting (shTRIM22) shRNAs (clones B7 to B9). **(A)** qRT-PCR quantitation of *TRIM22* mRNA levels in MRC5t shCtrl and shTRIM22 cells. Values normalized to shCtrl; n=3, means and SD shown. **(B)** MRC5t shCtrl and shTRIM22 (B7) cells were treated with (+) or without (-) IFN-β (100 IU/ml) for 24 h. WCLs were analysed by western blotting for TRIM22 expression. Actin is shown as a loading control. **(C, D)** MRC5t shCtrl or shTRIM22 (B7) cells were infected with a panel of human (WSN, A/Puerto Rico/8/1934(H1N1); PR8, and A/Udorn/307/1972(H3N2); Udorn) or avian (A/Duck/Singapore/5/1997(H5N3); Duck and A/Chicken/Italy/1067/1999(H7N1); Chicken) IAVs at a MOI of 0.05 FFU/cell (based on MRC5t titres) for 8h. **(C)** Representative confocal micrographs of PR8 infected MRC5t shCtrl or shTRIM22 cells. TRIM22 and IAV NP were labelled by indirect immunofluorescence. Nuclei were DAPI stained. **(D)** Relative (Rel.) fold increase in the number of NP antigen positive cells (shTRIM22/shCtrl) in IAV infected MRC5t cell monolayers. n ≥ 3, all data points shown; line, mean; whisker, SD. **(E)** Representative immunocytochemistry images of IAV (WSN; 50-100 PFU/monolayer) plaque formation (NP staining) in MRC5t shCtrl and shTRIM22 cell monolayers. **(F)** Relative fold increase in IAV plaque formation efficiency (PFE; shTRIM22/shCtrl) in MRC5t infected monolayers (as in **E**). n ≥ 3, all data points shown; line, mean; whisker, SD. **(D, F)** One sample two-tailed t test against a theoretical mean of 1; p-values shown. **(G)** Quantitation of relative IAV (WSN) PFE (shTRIM22/shCtrl) in cell monolayers treated with Ruxolitinib (Ruxo; 4 µM) or DMSO (carrier control). Values normalized to infected shCtrl cell monolayers treated with DMSO (dotted line). All data points shown; line, mean; whisker SD. Upper, two-tailed t test; lower, one sample two-tailed t test against a theoretical mean of 1; p-values shown.

As TRIM22 has been reported to act as a direct antiviral effector protein of IAV infection through the targeted ubiquitination and proteasome-dependent degradation of NP (Di Pietro et al., 2013; Pagani et al., 2018), we reasoned that the constitutive expression of this antiviral effector protein might provide cells with an immediate level of protection against infection. We therefore sought to explore the restrictive potential of cells that constitutively expressed TRIM22 to restrict IAV replication. We generated stable MRC5t cell lines expressing non-targeting control and TRIM22-targeting short hairpin RNAs (shCtrl and shTRIM22, respectively) sufficient to knockdown TRIM22 expression with or without IFN-β stimulation (**Figures 4A, B**). To test our hypothesis, we infected shCtrl or shTRIM22 MRC5t cells with a range of human (WSN; A/PR8/1934(H1N1), PR8; or A/Udorn/1972(H3N2), Udorn) or avian strains (A/Duck/Singapore/5/97(H5N3), Duck; or A/Chicken/Italy/1067/99(H7N1), Chicken) of IAV and examined the number of NP positive cells by indirect immunofluorescence at 8 hpi (**Figures 4C, D**). Depletion of TRIM22 significantly increased the number of IAV NP positive cells relative to infected control cells for the majority of strains examined. Correspondingly, we observed a significant increase in the number of IAV (WSN) plaques in MRC5t cells depleted of TRIM22 relative to control cells at 36 hpi under equivalent infection conditions (**Figures 4E, F**). This increase in plaque formation occurred independently of IFN signalling, as the addition of the JAK-STAT inhibitor Ruxolitinib (Ruxo), which effectively blocked the induction of ISG expression (*Mx1* and *ISG15*) following IFN stimulation (**Figure S1C**), had no effect on IAV plaque number in MRC5t cells [**Figure 4G**; (Stewart et al., 2014)]. Thus, pre-existing levels of TRIM22 expression are sufficient to restrict the initiation of IAV infection independently of the IFN response.

**Figure 5:**
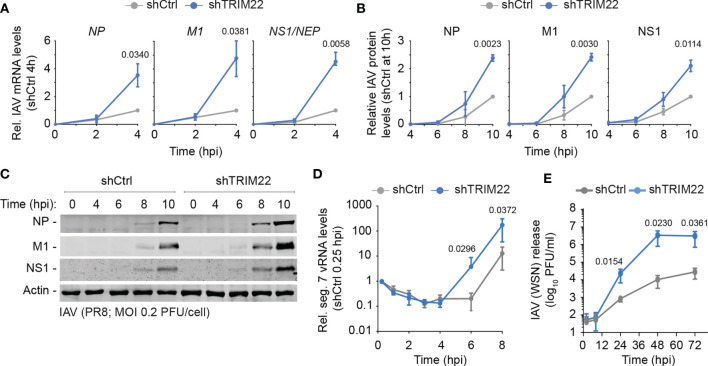
Constitutive TRIM22 expression restricts the onset of IAV transcription. MRC5t shCtrl and shTRIM22 cells were infected with IAV (PR8) at a MOI of 0.2 **(A–C)** or 0.05 **(D)** PFU/cell (based on MRC5t titres) and harvested at the indicated time points. **(A)** qRT-PCR quantitation of IAV *NP*, *M1*, and *NS1/NEP* mRNA levels. Values normalized to infected shCtrl samples at 4 hpi. n=3, means and SD shown. **(B, C)** Quantitation of viral protein (NP, M1, NS1) expression derived from western blots of infected WCLs. Actin is shown as a loading control. Values normalized to actin and expressed relative to levels in infected shCtrl cells at 10 hpi. n=3, means and SD shown. **(A, B)** One sample two-tailed t test against a theoretical mean of 1; p-values shown. **(D)** Quantification of IAV vRNA seg. 7 levels by qRT-PCR. Values normalized to infected shCtrl samples at 0.25 hpi. n=3, means and SD shown. **(E)** MRC5t shCtrl and shTRIM22 cells were infected with IAV (WSN) at a MOI of 0.001 PFU/cell (based on MRC5t titres). Media were harvested at the indicated time points and IAV plaque titre determined on MDCK cells; n=3, means and SD shown. **(D, E)** ratio paired two-tailed t test; p-values shown.

In order to investigate the kinetics of TRIM22 restriction further, we examined the point in the IAV replicative cycle at which it acted. We infected TRIM22 depleted or control MRC5t cells with IAV (PR8; MOI 0.2 PFU/cell) and harvested infected cell extracts over a range of early time points covering the initiating cycle of IAV infection. We used qRT-PCR and western blotting to measure viral transcription and protein expression of three gene products (NP, M1 and NS1) encoded by independent genome segments. TRIM22 depletion increased transcription of all three genes (**Figure 5A**), correlating in each case with an increase in viral protein synthesis (**Figures 5B, C**). Correspondingly, we could observe an enhanced rate of *de novo* vRNA synthesis by 6 hpi (**Figure 5D**). While transcription and replication of the IAV genome are intimately linked, the differences in mRNA levels were detectable prior to the differences observed in vRNA synthesis (4 hpi *vs* 8 hpi, respectively; **Figures 5A, D**). Thus, the suppression of viral transcription by TRIM22 is likely responsible for, or contributes to, the attenuation of IAV genome replication. To assess the impact of TRIM22 on the spread of infection, we infected TRIM22 depleted or control cells with IAV analysed the production of infectious virus released from infected cells over a time-course of infection. We observed an increase in infectious virus produced in TRIM22 depleted cells compared to control cells, confirming TRIM22-mediating restriction is sufficient to attenuate the replication and spread of IAV.

We conclude that constitutive levels of TRIM22 expression confer a pre-existing level of antiviral defence to IAV infection, which acts to supress IAV transcription within the first 4 hours of infection prior to detectible levels of ISG expression and independently of IFN-mediated innate immune defences (**Figures 3E**, **4G**).

### Laboratory-Adapted Cell Lines Demonstrate Alternate Patterns of Immune Gene Expression Relative to Bronchial Epithelial Cells

**Figure 6:**
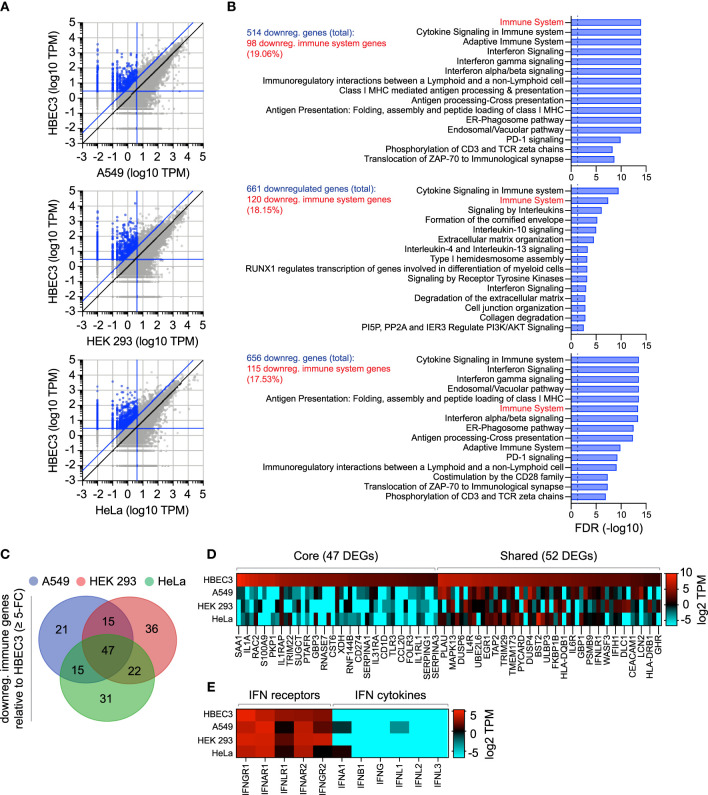
Laboratory-adapted cell lines have downregulated levels of constitutive immune gene expression relative to human bronchial epithelial cells. **(A)** Scatter plots showing differentially expressed gene (DEG) transcripts identified to be downregulated (blue circles; ≥ 5-fold change, diagonal blue line) in A549, HEK 293, and HeLa (top, middle, and bottom panels, respectively; vertical blue line, ≤ 4 TPM) relative to HBEC3 cells (horizontal blue line, ≥ 3 TPM). Values shown in **Supplemental Table S3A**. **(B)** Reactome pathway analysis of mapped DEGs (identified in **A**). The top 15 most significantly enriched pathways plotted (-log10 FDR shown; **Supplemental Tables S3B-D**). Dotted line, threshold of significance (-log10 FDR of 0.05). The number of downregulated DEGs identified and immune system percentile shown (blue and red text, respectively). **(C)** Venn diagram showing the number of unique and shared immune system DEGs (identified in **B**; **Supplemental Table S3E**) for each paired condition analyzed. **(D)** Heat map showing the expression profile (log2 TPM) of conserved (core, 47 genes) or shared (52 genes) immune system DEGs (as in **C**). Every second gene labelled. Values shown in **Supplemental Table S3F**. **(E)** Heat map showing IFN-related receptor and cytokine expression profiles (log2 TPM) in A549, HEK 293, HeLa, and HBEC3 cells. Values shown in **Supplemental Table S3G**.

As we had identified the constitutive expression of TRIM22 to occur in a tissue-specific and cell-type dependent manner (**Figures 1**–**3**), we next investigated if other antiviral regulators showed similar patterns of differential expression in laboratory-adapted cell lines. Although a functional characterization of these effector proteins is outside the scope of this study, we wished to establish whether the cell-type specific expression observed for TRIM22 was unique or more widely applicable. Using RNA-Seq data sets obtained from HPA [https://www.proteinatlas.org; (Uhlen et al., 2010; Uhlen et al., 2015)], we compared the transcript expression profile of human bronchial epithelial cells (HBEC3) to that of three laboratory-adapted cell lines (A549, HEK 293, and HeLa) widely used for respiratory virus research. For each pairwise comparison, we identified more than 500 differentially expressed genes (DEGs) to be downregulated (≥ 5-fold change) relative to HBEC3 cells (**Figures 6A, B** and **Supplemental Table S3A**). Pathway analysis identified a substantial proportion of these downregulated DEGs to map to the immune system in each case [**Figure 6B** and **Supplemental Tables S3B-D**); A549 (19.06%), HEK 293 (18.15%), and HeLa (17.53%)]. Out of the 187 unique immune genes identified to be downregulated relative to HBEC3 cells (**Supplemental Tables S3E, F**), 47 (25.1%) were downregulated in all three laboratory-adapted cell lines and 52 (27.8%) common to at least two cell lines (**Figures 6C, D**; core and shared DEGs, respectively). Notably, the identification of these DEGs occurred independently of detectible levels of IFN expression in HBEC3 cells (**Figure 6E** and **Supplemental Table S3G**), indicating that HBEC3 cells were not undergoing an innate immune response that would lead to elevated levels of immune gene expression at the point of RNA extraction. These data demonstrate that laboratory-adapted cell lines to have downregulated patterns of constitutive immune gene expression relative to bronchial epithelial cells, with many immune genes being downregulated in more than one cell type.

**Figure 7:**
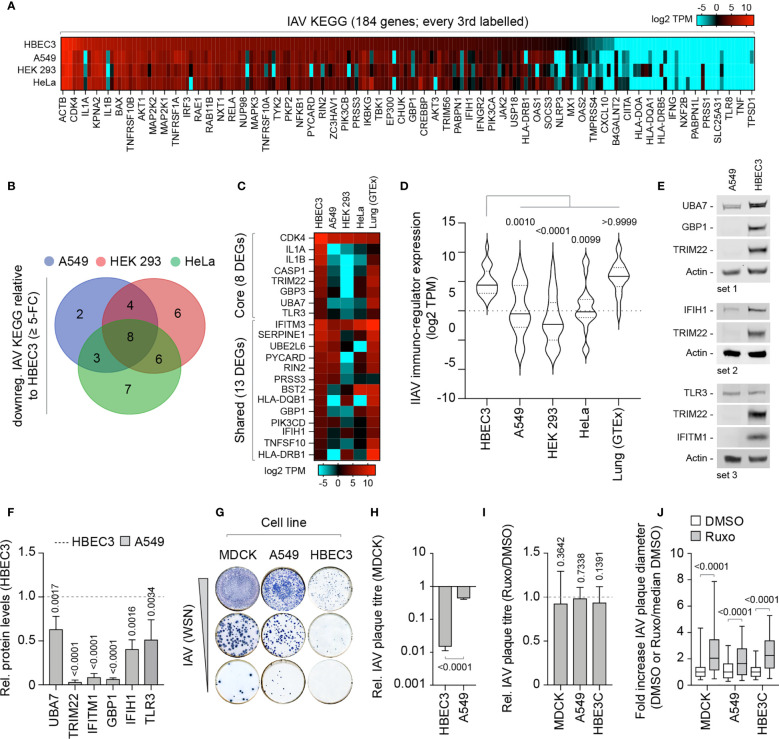
Laboratory-adapted cell lines show enhanced permissivity to IAV infection. **(A)** Heat map showing the expression profile (log2 TPM) of genes associated with an extended IAV KEGG network [hsa05164; (Villalon-Letelier et al., 2017)] in HBEC3, A549, HEK 293, and HeLa cells. Every second gene labelled. Values shown in **Supplemental Table S4A**. **(B)** Venn diagram showing the number of unique and shared DEGs identified to be downregulated (≥ 5-fold change) in A549, HEK 293, and HeLa cells (≤ 4 TPM) relative to HBEC3 cells (≥ 3 TPM per gene) for each paired condition analyzed. **(C)** Heat map showing expression profile (log2 TPM) of conserved (core, 8 genes) and shared (13 genes) DEGs identified to be downregulated in A549, HEK 293, and HeLa cells relative to HBEC3 cells (as in **B**) and associated values in lung tissue (GTEx, n=427). Values shown in **Supplemental Table S4B**. **(D)** Violin plot showing the expression profile of downregulated IAV KEGG DEGs (as in **C**). Horizontal solid lines; median gene expression. Horizontal dotted lines; 5^th^ and 95^th^ percentile range. Paired one-way ANOVA (Friedman multiple comparison test); p-values shown. **(A–D)** Data adapted under creative commons licence from HPA and GTEx portal, respectively (Uhlen et al., 2010; Carithers et al., 2015; Uhlen et al., 2015). **(E)** Western blots of WCLs derived from non-treated HBEC3 and A549 cells showing UBA7, GBP1, IFIH1, TLR3, IFITM1, and TRIM22 (+ve control) protein expression levels. Actin is shown as a loading control. **(F)** Quantitation of protein expression levels (as in **E**). Values normalized to actin and expressed relative to levels in HBEC3 cells (Dotted line). n≥3, means and SD shown. One-sample two-tailed t test against a hypothetical mean of 1; p-values shown. **(G)** Representative immunocytochemistry images of IAV plaque formation (NP staining) in MDCK, A549, and HBEC3 cells infected with equivalent serial dilutions of IAV (WSN). **(H)** Quantitation of plaque numbers in each cell line expressed relative (rel.) to MDCK cells (rel. plaque titre); n≥3, means and SD shown. Mann-Whitney U-test; p-value shown. **(I)** MDCK, A549, and HBEC3 cells were pre-treated for 1 h in the presence of Ruxolitinib (Ruxo; 5 µM) or DMSO (carrier control) prior to infection with serial dilutions of IAV (WSN) in the presence of drug or carrier control. Quantitation of rel. plaque titre (plaque titre with Ruxo/plaque titre with DMSO) for each cell line is shown. n≥3, means and SD shown. One-sample two-tailed t test against a hypothetical mean of 1; p-values shown. **(J)** Quantitation of the fold increase in IAV plaque diameter in Ruxo or DMSO-treated infected cell monolayers (as in **I**). Values normalized to the median DMSO plaque diameter in each cell line (DMSO or Ruxo/median DMSO). n≥3, means and SD shown. Mann-Whitney U-test; p-values shown.

We next investigate if these alternate patterns of immune gene expression could potentially influence the outcome of IAV infection. We curated an extended IAV KEGG network ([hsa05164]) to include recently identified restriction factors [**Supplemental Table S4A**; (Shim et al., 2017; Villalon-Letelier et al., 2017)]. Out of the 184 genes analyzed, 36 (19.5%) were identified to be downregulated (≥ 5-fold change) in at least one cell line relative to HBEC3 cells (**Figure 7A**). Out of these, 8 were downregulated in all three cell lines and 13 common to at least two cell lines (**Figures 7B, C**). Importantly, the basal levels of gene expression observed for these DEGs in HBEC3 cells were similar to those observed in lung tissue (**Figures 7C, D** and **Supplemental Table S4B**), indicating that these host factors were downregulated in laboratory-adapted cell lines relative to both bronchial epithelial cells and lung tissue. We next examined the protein expression profile of a subset of these DEGs (TRIM22, UBA7, IFITM1, GBP1, and IFIH1) by western blotting. In each instance, significantly lower levels of protein expression were detected in A549 cells relative to HBEC3 cells (**Figures 7E, F**), confirming cell-type dependent patterns of constitutive immune gene expression. We hypothesized that such alternate patterns of immune gene expression may influence the initiation or outcome of IAV infection in a cell-type dependent manner. To examine this, we compared the relative plaque titre of IAV in HBEC3 and A549 cells to that of MDCK cells. Consistent with their pattern of immune gene expression, HBEC3 cells were highly restrictive to IAV plaque formation relative to both MDCK (≥ 70-fold) and A549 (≥ 30-fold) cells under equivalent infection conditions (**Figures 7G, H**). This cell-type dependent increase in IAV restriction occurred independently of IFN-mediated innate immune defences, as the addition of Ruxolitinib did not affect IAV plaque number in any of the cell types examined [**Figure 7I**; (Stewart et al., 2014)]; although an increase in relative plaque size could be observed in each instance (**Figure 7J**). Thus, we identify alternate patterns of pre-existing immune gene expression to correlate with cell line permissivity to IAV infection. Importantly, we show these pre-existing levels of antiviral gene expression to be significantly downregulated in many laboratory-adapted cell lines commonly used for respiratory virus research relative to HBEC3 cells or lung tissue, the natural site of infection. Whether there is a common cause responsible for this loss of pre-existing antiviral gene expression remains an interesting question for future research.

## Discussion

We hypothesized that tissues exposed to the constant threat of infection may constitutively express higher levels of pre-existing immune factors to reduce the need to activate potentially harmful innate immune defences, thereby creating localized patterns of antiviral protection. Here we identify lung tissue to have enriched levels of constitutive ISG expression relative to other mucosal and non-mucosal tissues (**Figure 1**). We identify the antiviral host factor TRIM22 to be one of several antiviral proteins to be constitutively expressed in lung tissue and primary cells of lung origin independently of immune stimulation or IAV infection (**Figure 2**). We show that the constitutive expression of TRIM22 provides an element of pre-existing antiviral defence to IAV infection independently of IFN-mediated innate immune defences (**Figures 3**, **4**).

Our finding that TRIM22 is one of the most abundantly expressed TRIM proteins in lung tissue came as a surprise (**Figures 1**, **2**), as the expression of TRIM22 has been widely reported to require viral infection or immune stimulation in multiple cell culture model systems [**Figure 3** and **Figure S1**; (Hattlmann et al., 2012; Di Pietro et al., 2013)]. Although we do not know the genetic or biological causes responsible for the differential patterns of TRIM22 expression observed, we note that TRIM22 (formerly known as Staf50) has been shown to be upregulated by other cytokines, including interleukins-1β, -2 and -15, progesterone, and tumour necrosis factor-α (Hattlmann et al., 2012). Tonic IFN-signalling by host microbiota may also contribute to the high levels of TRIM22 expression observed in lung tissue (**Figures 1**, **2**), as the antibiotic treatment of animals has been shown to influence lung ISG expression levels in an IFN-receptor dependent manner (Bradley et al., 2019; Stefan et al., 2020). However, the presence of host microbiota *in vivo* does not account for the cell-type dependent profiles of TRIM22 or ISG expression observed between cultured cells (**Figures 3**, **6**, and **7**), which indicate an additional level of genetic regulation. Notably, TRIM22 expression has been shown to be regulated by p53 and found to correlate with cellular differentiation and proliferation status (Obad et al., 2004; Hattlmann et al., 2012). Thus, the downregulation of TRIM22 expression in many laboratory-adapted cell lines may occur due to alternative patterns of cytokine expression, p53 inactivation, and/or sustained cellular proliferation in a cell-type dependent manner. It is also evident that many carcinoma cell lines carry variable gene copy numbers and single nucleotide polymorphisms (SNPs) which can influence protein expression levels and immune competence (Barretina et al., 2012; Frishberg et al., 2019; Ghandi et al., 2019). Indeed, Cancer Cell Line Encyclopaedia [CCLE; (Barretina et al., 2012; Ghandi et al., 2019)] records demonstrate TRIM22 transcript levels and copy number are downregulated in many lung carcinoma cell-types (https://portals.broadinstitute.org/ccle/page?gene=TRIM22). We note that transformed cell lines which lack constitutive TRIM22 expression include many model cell lines currently used for respiratory virus research. As intrinsic immunity contributes to the resistance of cells to infection (Bieniasz, 2004; Komatsu et al., 2016; Wu et al., 2018; Alandijany, 2019; Bergantz et al., 2019), it seems plausible that adoption of cell types suited to high levels of virus propagation may have unwittingly selected for cells that fail to express one or more intrinsic antiviral effector molecules. Such differences may therefore account for some of the experimental variation debated in the scientific literature between cell culture systems.

Previous reports have demonstrated that TRIM22 can function as a direct-acting antiviral effector protein against a wide variety of DNA and RNA pathogens (Barr et al., 2008; Eldin et al., 2009; Gao et al., 2009; Hattlmann et al., 2012; Di Pietro et al., 2013; Yang et al., 2016). In the context of IAV, the IFN-stimulated induction of TRIM22 has been shown to mediate IAV restriction in A549 cells through the ubiquitination of NP leading to its proteasome-dependent degradation (Di Pietro et al., 2013; Pagani et al., 2018). NP is an essential component of vRNPs, required for viral gene expression, genome replication, and *de novo* vRNP assembly (Eisfeld et al., 2015; Hutchinson, 2018). Our data corroborate that TRIM22 functions as an antiviral effector against IAV infection, likely enacted through its ability to target NP for degradation as previously reported (Di Pietro et al., 2013; Pagani et al., 2018). However, unique from its reported role as an effector protein of the IFN-response, we demonstrate that pre-existing levels of constitutive TRIM22 expression are sufficient to restrict the onset of IAV transcription from the outset of infection (**Figure 5A**).

We propose that the pre-existing antiviral defence conferred by constitutive expression of TRIM22 may play an important role in protecting the host during the initial exposure of respiratory cells to invading virus, prior to the spread of infection and activation of the innate immune response. Our observation that TRIM22 restricts the number of productive infectious events following IAV challenge suggests that this pre-existing defence can influence the outcome of infection in individual cells (**Figure 4**). This may be particularly relevant when host tissues are exposed to low levels of virus challenge. Our observation that constitutive TRIM22 also restricts the spread of virus infection suggests that this pre-existing defence may also play a valuable role in slowing down the rate of IAV replication prior to activation of additional host defences (**Figure 5E**). The presence of such pre-existing antiviral defences is likely to provide additional advantages over pathogen-induced immunity. Firstly, many viruses encode antagonists of immune signalling that can inhibit or attenuate the induction of host immune responses (Hale et al., 2010; Hsu, 2018). Thus, in the evolutionary arms race between virus and host, the constitutive expression of key antiviral factors may provide a valuable initial defence that cannot be counteracted by simply supressing host transcription. Secondly, the ability to immediately limit infection may reduce the need to upregulate potentially harmful pro-inflammatory immune responses that can themselves contribute to host pathology, as is the case for severe IAV respiratory disease and ARDS (acute respiratory distress syndrome) following SARS-CoV-2 (severe acute respiratory syndrome coronavirus 2) infection (La Gruta et al., 2007; Iwasaki and Pillai, 2014; Liu et al., 2016; Wu et al., 2020; Zhu et al., 2020).

We propose that TRIM22 likely functions along with other known constitutively expressed antiviral effector proteins, for example TRIM32 and TRIM41, which have been shown to have intrinsic antiviral functions against IAV (Fu et al., 2015; Patil et al., 2018). The identification of additional ISGs, including BST2, IFITM1, SAMHD1, and GBP family members, that demonstrate tissue-specific and cell-type dependent (**Figures 1**, **7**) patterns of constitutive expression raises the possibility that other antiviral proteins may also contribute to this pre-existing defence. Given that TRIM22 is reported to restrict multiple different viruses, it is tempting to speculate that pre-existing levels of TRIM22 expression might also be relevant to the control of other important respiratory viruses. Thus, we anticipate that our findings will provide a foundation for future work that could further develop the concept of intrinsic immunity within cells of the respiratory airway.

In conclusion, we identify tissue-specific and cell-type dependent patterns of constitutive TRIM22 expression in the respiratory tract to confer a pre-existing intracellular defence to IAV replication. We show TRIM22 to be among many antiviral effector molecules upregulated at this portal of viral entry and propose that the importance of such constitutive levels of antiviral gene expression in the control of respiratory infections has yet to be fully appreciated.

## Data Availability Statement

The datasets generated and analysed in this study are available in the **Supplemental Tables S1** to **S4** and **Supplemental File S1** in **Supplementary Material**.

## Ethics Statement

No animals were directly subjected to experimentation as part of this scientific study. All animal tissues were obtained from previous material with permission from Public Health England (PHE). Procedures associated with this earlier study were approved by the PHE Ethical Review Committee (Porton Down, UK), authorized under UK Home Office project licence 30/3083, and performed according to UK Animal (Scientific procedures) Act 1986.

## Author Contributions

MCh: Conceptualization, Data curation, Formal Analysis, Investigation, Methodology, Validation, Visualization, Writing – original draft, Writing – review & editing. SM: Formal Analysis, Investigation, Resources, Methodology, Supervision. JW: Data curation, Formal Analysis, Investigation, Methodology, Validation. ES: Data curation, Formal Analysis, Investigation, Methodology, Validation. RD: Formal Analysis, Investigation, Methodology, Validation. GL: Formal Analysis, Investigation, Methodology, Validation. MA-S: Formal Analysis, Investigation, Methodology, Validation. LH: Formal Analysis, Investigation, Methodology, Validation. MCa: Data curation, Formal Analysis, Funding acquisition, Supervision. JS: Data curation, Formal Analysis, Funding acquisition, Supervision. PD: Funding acquisition, Supervision, Writing – review & editing. EH: Conceptualization, Funding acquisition, Methodology, Project administration Supervision, Validation, Writing – Original draft, Writing – review & editing. CB: Conceptualization, Funding acquisition, Methodology, Project administration Supervision, Validation, Writing – Original draft, Writing – review & editing. All authors contributed to the article and approved the submitted version.

## Funding

MCh, SM, and CB were funded by the UK Medical Research Council (MRC; MC_UU_12012/5 and MC_UU_12014/5) https://mrc.ukri.org/ awarded to CB. ES and EH were funded by the MRC (MR/N008618/1) awarded to EH. RD and PD were funded by the Biotechnology and Biological Sciences Research Council (BBSRC; BB/J004324 and BB/K012681/1) https://bbsrc.ukri.org. MA-S and JS were funded by the BBSRC (BB/K009664/1, BB/R00904X/1, and BB/R01863/1). The funders had no role in the study design, data collection and analysis, decision to publish, or preparation of the manuscript.

## Conflict of Interest

The authors declare that the research was conducted in the absence of any commercial or financial relationships that could be construed as a potential conflict of interest.

## Publisher’s Note

All claims expressed in this article are solely those of the authors and do not necessarily represent those of their affiliated organizations, or those of the publisher, the editors and the reviewers. Any product that may be evaluated in this article, or claim that may be made by its manufacturer, is not guaranteed or endorsed by the publisher.

## Supplementary Material

Supplementary Table 1 Constitutive ISG and IFN-related transcript expression levels across a range of human tissues. **(S1A)** Transcript expression values (log2 TPM; 0 = 0.01) of 200 ISGs [previously shown to be upregulated ≥ 8-fold change in response to universal IFN treatment; (Shaw et al., 2017)] across a range of mucosal (lung, small intestine [int.; terminal ileum], esophagus [mucosa], colon [sigmoid]), and non-mucosal (liver, skin [suprapubic], and kidney [cortex] tissues. **(S1B)** Transcript expression values (log2 TPM; 0 = 0.01) of IFN-related receptors and cytokines across human tissues (as in **S1A**). Data adapted under creative commons license from Human Protein Atlas [HPA; (Uhlen et al., 2010; Uhlen et al., 2015)] and Genotype-Tissue Expression [GTEx; (Carithers et al., 2015)] project.

Supplementary Table 2 TRIM family member transcript expression values across a range of human tissues. **(S2A)** Transcript expression values of 67 TRIM family member in human lung tissue. **(S2B)** TRIM22 transcript expression values across a range of human tissues. Data adapted under creative commons license from Human Protein Atlas [HPA; (Uhlen et al., 2010; Uhlen et al., 2015)] and Genotype-Tissue Expression [GTEx; (Carithers et al., 2015)] project.

Supplementary Table 3 Constitutive levels of antiviral gene expression are downregulated in laboratory-adapted cell lines relative to human bronchial epithelial cells. **(S3A)** Transcript expression values (log2 TPM) for differentially expressed genes (DEGs) identified to be downregulated (≥ 5-fold change) in A549, HEK 293, and HeLa cells (≤ 4 TPM) relative to HBEC3 cells (≥ 3 TPM; blue circles in **Figure 6A**). **(S4B-D)** Reactome (https://reactome.org) pathway analysis of mapped DEGs (as in S4A; blue circles in **Figure 6A**) identified to be downregulated for each pairwise comparison [A549 **(S4B)**, HEK 293 **(S4C)**, and HeLa **(S4D)**] relative to HBEC3 cells. **(S4E)** Identification of conserved (core), shared, or unique immune system DEGS downregulated in A549, HEK 293, and HeLa cells relative to HBEC3 cells. **(S4F)** Transcript expression values (log2 TPM) of core and shared DEGs downregulated in A549, HEK 293, and HeLa cells relative to HBEC3 cells. **(S4G)** Transcript expression values (log2 TPM) of IFN-related receptors and cytokines in A549, HEK 293, HeLa and HBEC3 cells. Data adapted under creative commons license from Human Protein Atlas [HPA; (Uhlen et al., 2010; Uhlen et al., 2015)].

Supplementary Table 4 Extended IAV KEGG network transcript expression values across a range of cell lines and lung tissue. **(S4A)** Transcript expression values (log2 TPM) of 184 IAV genes (extended (ext.) IAV KEGG network [hsa05164; (Villalon-Letelier et al., 2017)] in HBEC3, A549, HEK 293, and HeLa cells. **(S4B)** Transcript expression values (log2 TPM) of DEGs identified to be downregulated (≥ 5-fold change) in A549, HEK 293, and HeLa cells (≤ 4 TPM) relative to HBEC3 cells (≥ 3 TPM) and equivalent expression values in human lung tissue (GTEx, n=427). Data adapted under creative commons license from Human Protein Atlas [HPA; (Uhlen et al., 2010; Uhlen et al., 2015)] and Genotype-Tissue Expression [GTEx; (Carithers et al., 2015)] project.

Supplementary Table 5 Custom IAV primer-probe sequences. Nucleotide sequences for custom IAV (*NP*, *M1*, *NS1*/*NEP*) primer-probes mixes used in the study.

Supplemental File S1 Raw data for line drawings presented in this study.
