# Supplementary figures and images for: Constitutive TRIM22 Expression in the Respiratory Tract Confers a Pre-Existing Defence Against Influenza A Virus Infection
Source: Front Cell Infect Microbiol. 2021 Sep 21;11:689707. doi: 10.3389/fcimb.2021.689707 (PMC8490869; doi:10.3389/fcimb.2021.689707)

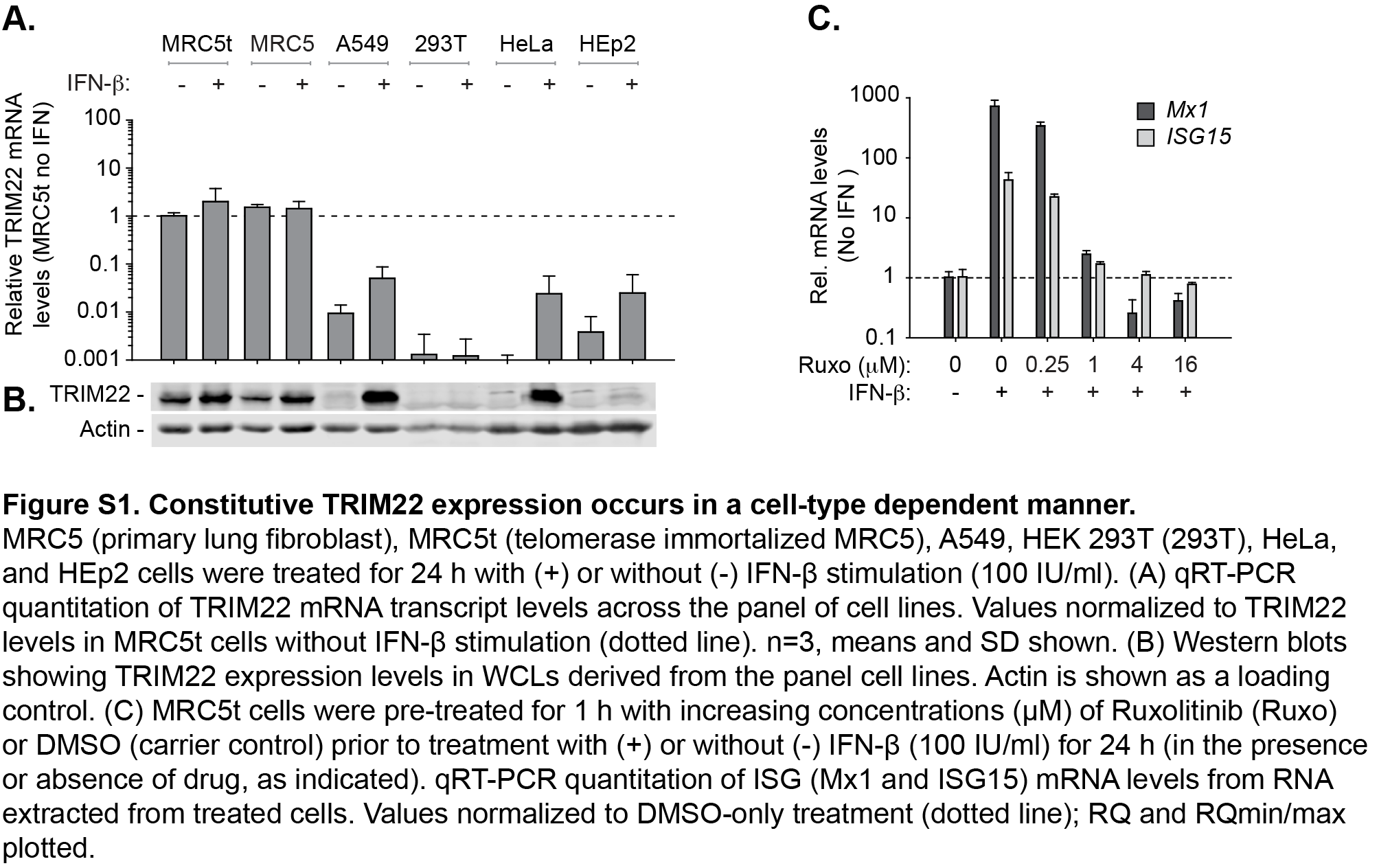

Supplement: Supplementary Table 1 — Constitutive ISG and IFN-related transcript expression levels across a range of human tissues. (S1A) Transcript expression values (log2 TPM; 0 = 0.01) of 200 ISGs [previously shown to be upregulated ≥ 8-fold change in response to universal IFN treatment; (Shaw et al., 2017)] across a range of mucosal (lung, small intestine [int.; terminal ileum], esophagus [mucosa], colon [sigmoid]), and non-mucosal (liver, skin [suprapubic], and kidney [cortex] tissues. (S1B) Transcript expression values (log2 TPM; 0 = 0.01) of IFN-related receptors and cytokines across human tissues (as in S1A). Data adapted under creative commons license from Human Protein Atlas [HPA; (Uhlen et al., 2010; Uhlen et al., 2015)] and Genotype-Tissue Expression [GTEx; (Carithers et al., 2015)] project. [file DataSheet_1.zip › Supplemental figure 1.TIF]
